# Molecular characterization of human T-cell leukemia virus type 1 gp46 glycoprotein from healthy carriers and tropical spastic paraparesis/HTLV-associated myelopathy infected individuals

**DOI:** 10.1186/1742-4690-11-S1-P83

**Published:** 2014-01-07

**Authors:** Aline CA Mota-Miranda, Fernanda K Barreto, Maria FC Amarante, Everton Baptista, Joana P Monteiro-Cunha, Lourdes Farre-Valve, Bernardo Galvao-Castro, Luiz CJ Alcantara

**Affiliations:** 1 CPqGM/FIOCRUZ, Salvador, Bahia, Brazil; 2 EBMSP, Salvador, Bahia, Brazil; 3 UFBA, Salvador, Bahia, Brazil; 4 NCI, NIH, Bethesda, MD, USA

## 

The present study attempts to assess the molecular diversity of gp46 glycoprotein in HAM/TSP patients and Health Carrier (HC) individuals. The median HTLV-1 proviral load of HC (n=5) and HAM/TSP (n=5) patients was similar (average 316,227 copies/106 PBMCs). The gp46 molecular characterization of 146 clones (70 HC and 76 HAM/TSP) revealed an overall diversity, within HC and HAM/TSP clones, of 0.4% and 0.6%, respectively. A single amino acid (aa) substitution (S35L) was exclusive for the HC group, and three gp46 substitutions (F14S, N42H, G72S) were exclusive for the HAM/TSP group. The remaining frequent mutation (V247I) was present in both groups (p = 0.0014). The in silico protein analysis revealed that the mutated alleles F14S and N42H represent more hydrophilic and flexible protein domains that are likely to be less antigenic. The Receptor Binding Domain is quite variable in the HAM/TSP group. Two other domains (aa 53-75 and 175-209) that contain multiple linear T-cell epitopes showed genetic diversity in both HAM/TSP and HC groups. Further analysis revealed 27 and 13 T-cell epitopes for class I HLA alleles and class II HLA alleles, when analyzing the entire gp46. We still cannot associate any of the gp46 found mutations with the clinical profile.

